# Functional connectivity in Lewy body disease with visual hallucinations

**DOI:** 10.1111/ene.16115

**Published:** 2023-11-01

**Authors:** Michael J. Firbank, Daniel Collerton, Katrina daSilva Morgan, Julia Schumacher, Paul C. Donaghy, John T. O'Brien, Alan Thomas, John‐Paul Taylor

**Affiliations:** ^1^ Translational and Clinical Research Institute Newcastle University Newcastle upon Tyne UK; ^2^ Deutsches Zentrum für Neurodegenerative Erkrankungen Standort Rostock/Greifswald Rostock Mecklenburg‐Vorpommern Germany; ^3^ Department of Neurology University Medical Center Rostock Rostock Germany; ^4^ Department of Psychiatry, School of Clinical Medicine University of Cambridge Cambridge UK

**Keywords:** functional connectivity, Lewy body disease, MRI, visual hallucinations

## Abstract

**Background and purpose:**

Visual hallucinations are a common, potentially distressing experience of people with Lewy body disease (LBD). The underlying brain changes giving rise to visual hallucinations are not fully understood, although previous models have posited that alterations in the connectivity between brain regions involved in attention and visual processing are critical.

**Methods:**

Data from 41 people with LBD and visual hallucinations, 48 with LBD without visual hallucinations and 60 similarly aged healthy comparator participants were used. Connections were investigated between regions in the visual cortex and ventral attention, dorsal attention and default mode networks.

**Results:**

Participants with visual hallucinations had worse cognition and motor function than those without visual hallucinations. In those with visual hallucinations, reduced functional connectivity within the ventral attention network and from the visual to default mode network was found. Connectivity strength between the visual and default mode network correlated with the number of correct responses on a pareidolia task, and connectivity within the ventral attention network with visuospatial performance.

**Conclusions:**

Our results add to evidence of dysfunctional connectivity in the visual and attentional networks in those with LBD and visual hallucinations.

## INTRODUCTION

Individuals with Lewy body disease (LBD) often experience complex visual hallucinations, for example of people and animals [1, 2]. These impact on day‐to‐day function and can cause significant distress to both patients and caregivers [3].

The exact aetiology of visual hallucinations in LBD is not understood, although several models have been suggested [4, 5]. Common elements in these models include a breakdown in communication between cortical regions involved in visual processing, in particular dysfunctional visual sensory information processing and distorted visual information outflow from the primary visual cortex to the temporal and frontal lobes. Deficits in attention, including reduced attentional guidance of sensory data gathering and imprecise processing of already gathered data, lead to a greater weighting of expected perceptions over sensory input and hence a bias towards internal mental imagery.

Predictions about specific brain network changes are made by two of the visual hallucinations models—the Attentional Networks (AN) model of Shine et al. [6, 7] and the Thalamocortical Dysrhythmia Default Mode Network Decoupling (TDDMN) model of Onofrj et al. [8].

A recent review of functional magnetic resonance imaging (fMRI) [9] in hallucinations concluded there was evidence for increased functional connectivity within the default mode network (DMN) and ventral attention network (VAN), decreases within the dorsal attention network (DAN), along with increased DMN to visual network connections and decreased connectivity between the DAN and both the VAN and DMN. The authors noted, however, the relative lack of studies, and the typically small number of participants; nevertheless, the results are broadly in keeping with the AN model which suggests that, along with reduced visual sensory processing, there is upregulation of VAN and DMN, a lack of integration of these with the exogenous coordination DAN, and increased connectivity of DMN and the visual network—a combination which leads to an overreliance on autobiographical priors and, subsequently, visual hallucinations. The fMRI connectivity evidence is also partially supportive of the TDDMN model, which posits inhibition of all attentional networks (in contrast to the upregulated VAN of the AN model) and leads to decoupling of the DMN, which in turn causes an increased influence of autobiographical priors.

In this paper, it was sought to utilize resting state fMRI to determine the strength of functional connectivity between the VAN, DAN, DMN and visual networks in LBD. It was further sought to use dynamic causal modelling (DCM) to characterize the causal relationships between any networks altered in people with LBD in relation to visual hallucinations. As noted by Uddin et al. [10] the terms VAN, salience network and cingulo‐opercular network have all been used to describe a functional brain network that includes the anterior insula, with the broad cognitive role of identifying salient information. For consistency with previous research in visual hallucinations the term VAN will be used here. Based on previous findings and model predictions, it is hypothesized that there is

1. an increase in connectivity within the VAN and DMN networks;
2. increased connectivity between the VAN and DMN and from the DMN to the visual network;
3. decreased directed connectivity of the DAN to other regions.

## METHODS

### Subjects

Data for this analysis were taken from several previous cohort studies at Newcastle: 32 with dementia with Lewy bodies (DLB) and 10 healthy controls from the AMPLE study [11], 34 with Parkinson's disease (PD) and 20 controls from VEEG‐Stim [12], and 38 with probable mild cognitive impairment with Lewy bodies (MCI‐LB) and 31 controls from the SUPERB study [13].

The DLB participants all met the current criteria for probable DLB [2], Parkinson's patients fulfilled the criteria for PD [14] and MCI‐LB patients met the research criteria for probable MCI‐LB [15]. Parkinson's patients were classed as having mild cognitive impairment according to the Movement Disorder Society level 1 criteria [16] or dementia according to the diagnostic criteria for PD dementia [17]. Control participants showed no evidence of cognitive decline and did not report having ever experienced visual hallucinations. All participants provided written informed consent, and the original studies were all approved by local ethics committees.

### Assessment

Participants underwent a detailed clinical assessment at baseline including physical and neurological examination. Informants were also interviewed if available to provide further information. Interviews for all studies included the Unified Parkinson's Disease Rating Scale (UPDRS) Part III, the Neuropsychiatric Inventory (NPI) and a visual angle discrimination test [18]. For VEEG‐Stim and SUPERB, the North‐East Visual Hallucinations Inventory (NEVHI) was also utilized alongside the noise pareidolia test [19] which contains cloud‐like noise images, including eight images in which different faces are inserted at varying locations. This test has been shown to discriminate those with a tendency to hallucinate in DLB [20].

Cognition was assessed using the Addenbrooke's Cognitive Examination Revised (ACE‐R) for AMPLE and SUPERB and the Cambridge Cognitive Examination (CAMCOG) for VEEG‐Stim. To allow comparison across studies, a global cognitive *Z* score was calculated for all participants from the mean and SD of the control group separately for the ACE‐R and CAMCOG tests. As a measure of visuospatial ability, the visuospatial score from ACE‐R and the praxis score from CAMCOG were used. To allow comparison across studies, these were divided by the maximum score (16 for ACE‐R and 12 for CAMCOG). Maximum scaling was used rather than *Z* score as there were ceiling effects in the control group.

The presence or absence of complex visual hallucinations was determined by two experienced old age psychiatrists based on clinical interview and a review of the patient notes and the NEVHI where available. Participants were thus split into control and Lewy body with (LB_VH) and without (LB_noVH) complex visual hallucinations.

### Imaging

All subjects were scanned with the same imaging sequences at the Newcastle Magnetic Resonance Centre. Imaging was performed on a 3 T Philips Intera Achieva scanner. Structural images were acquired with a magnetization prepared rapid gradient‐echo sequence, sagittal acquisition, echo time 4.6 ms, repetition time 8.3 ms, inversion time 1250 ms, flip angle 8°, SENSE factor 2, in‐plane field of view 240 × 240 mm with slice thickness 1.0 mm, yielding a voxel size of 1.0 × 1.0 × 1.0 mm [3].

Resting state scans were acquired with gradient‐echo echo‐planar imaging, repetition time 2072 ms, echo time 30 ms, SENSE factor 1.3; 64 × 63 matrix, field of view 192, 3 × 3 mm voxel size, with 33 slices of 3 mm (+1 mm gap), 290 dynamics, 10 min. Participants were asked to keep their eyes closed during the scan.

### Image processing

The Conn toolbox (version CONN21.a) with SPM (version 7771) default preprocessing pipeline was used to spatially normalize and motion correct the fMRI images and segment and normalize the T1 structural scans.

To identify regions for the connectivity analysis, the motion corrected images from Conn were processed in GIFT (https://trendscenter.org/software/gift/) to identify group‐specific networks using the independent component method with reference, which finds components utilizing an a priori template, for which the visual, DAN, VAN and DMN components of the seven network atlas described by Yeo et al. [21] were used. Nodes (8 mm radius spheres) of the networks were then identified from the maxima of the components based on the regions in Razi et al. [22]. The total number of regions was restricted to 15 to limit computational demand for the DCM analysis. Regions were visual, left and right occipital pole; DMN, posterior cingulate cortex, left and right inferior parietal, medial prefrontal cortex; DAN, bilateral parietal lobe and frontal eye fields; VAN, bilateral lateral parietal and anterior insula, anterior cingulate cortex (ACC). Location of the regions is shown in Table 1 and Figure S1.

**TABLE 1:** Location of the regions of interest used in the analysis.

Region	MNI coordinates (mm)		
Vis R occipital	7	−81	6
Vis L occipital	−7	−81	6
DMN PCC	0	−54	31
DMN R parietal	50	−58	27
DMN L parietal	−50	−62	28
DMN PFC	1	53	1
DAN R parietal	19	−66	57
DAN L parietal	−20	−66	57
DAN R FEF	27	0	56
DAN L FEF	−26	−2	57
VAN ACC	0	14	37
VAN R insula	36	14	6
VAN L insula	−36	14	6
VAN R parietal	60	−30	30
VAN L parietal	−60	−30	24

Abbreviations: ACC, anterior cingulate cortex; DAN, dorsal attention network; DMN, default mode network; FEF, frontal eye fields; L, left; PCC, posterior cingulate cortex; PFC, prefrontal cortex; R, right; VAN, ventral attention network; Vis, visual network.

For the (undirected) functional connectivity analysis these spherical regions of interest (ROIs) in the Conn toolbox were used, and region to region connectivity analysis was carried out using the default parameters, with scans denoised and outlier scans identified. The detected outlier scans were defined as covariates to eliminate movement artefacts. Confounds in the connectivity analysis were the default aCompCor (white and cerebrospinal fluid ROIs, five components each), scrubbing (as many regressors as identified invalid scans) and motion regression (12 regressors: six motion parameters + six first‐order temporal derivatives). A bandpass filter of 0.008–0.09 Hz was applied. Any participants with (a) fewer than 200 (out of 290) valid volumes or (b) mean framewise motion >0.4 mm or (c) any framewise motion >4 mm were excluded from analysis.

For DCM, the SPM volume of interest tool was used to extract the first eigenvector time course of each ROI for each subject from a general linear model including regressors of cerebrospinal fluid and white matter (obtained from the Conn segmentation masks) and the motion and scrubbing covariates from Conn.

Spectral DCM was performed with the DCM toolbox (version DCM12.5) in SPM. A fully connected model was specified for each subject and inverted to determine the connectivity matrix. The connectivity parameters were then extracted and analysed using linear models.

### Statistics

The (undirected) functional connectivity analysis was performed in Conn, using the default hierarchical clustering and cluster level inference using the functional network connectivity cluster threshold *p* < 0.05, cluster level *p* false discovery rate corrected (multivariate pattern analysis [MVPA] omnibus test) and connection threshold (*p* < 0.05, *p* uncorrected) to perform an *F* test for group difference. The connectivity matrix for each subject was also exported for analysis in R (version 4.0.4). For the significant clusters of connections identified in Conn, the connections for those ROIs from the DCM analysis were averaged, and linear regression was performed in R to compare groups.

For each of the five networks, the undirected and DCM connectivity parameters of the component ROIs were averaged to create a 5 × 5 connectivity matrix, and the DCM average self‐inhibitory connection was also calculated for the ROIs in each network. A linear model was used to compare connection strength between LB_VH and the other two groups for the network connections with hypothesized differences (within network connections for VAN, DAN, DMN; DAN to VAN and DMN; VAN to DMN, DMN to visual).

The Fisher exact test was used to compare sex between groups, ANOVA was used to compare continuous variables between all three groups, and *t* tests were used to compare between LB_noVH and LB_VH. *t* tests were done without assuming equal variance. Linear regression within the LB_VH group was used to investigate associations between significant connectivity clusters and the NPI hallucination subscore, the pareidolia noise task correct score, the angle test score and the visuospatial composite score.

## RESULTS

Twenty‐six participants (11 control, three DLB, four PD dementia, two PD with mild cognitive impairment, seven MCI‐LB) were excluded due to excess movement on the scan, leaving 41 with LB_VH, 48 with LB_noVH and 60 controls. Figure S1 shows the image quality control graphs. There were no group differences on maximum framewise motion (*F*_2,146_ = 1.2; *p* = 0.3) and mean motion (*F*_2,146_ = 0.35, *p* = 0.7), although the number of excluded volumes was significantly different between groups (*F*_2,146_ = 4.1, *p* = 0.018), being slightly higher in the LB_VH group (mean 9.4 volumes excluded vs. 4.5 for control and LB_noVH).

Table 2 shows the demographics and cognitive scores for the participants included in the analysis. Compared to LB‐noVH participants, LB_VH participants had significantly worse cognition and higher UPDRS motor score. As expected, LB_VH participants had a higher score on the NPI hallucination scale, worse ACE‐R visuospatial score and, on the pareidolia task, missed a higher proportion of faces which were present and tended to see faces which were not there. Table S1 includes a breakdown of participants according to diagnosis (DLB, PD with mild cognitive impairment, PD dementia, MCI‐LB). Table S1 includes details of cholinesterase inhibitor, antipsychotic and parkinsonian medication. There were no significant differences in medication between those with versus without visual hallucinations in the participants with either dementia or mild cognitive impairment. Of those with visual hallucinations who completed the NEVHI, 18/22 had experienced visual hallucinations in the last month, 2/22 within the last year and one participant last experienced visual hallucinations over a year ago. Table S3 includes the details of the hallucinations as recorded using the NEVHI.

**TABLE 2:** Participants included in the analysis: apart from age, all statistical comparisons are between LB_noVH and LB_VH.

	Control (60)	LB_noVH (48)	LB_VH (41)	Statistics
Age	74.7 (6.6) [61:92]	74.2 (6.8) [60:87]	75.8 (5.5) [62:89]	*F*_2,146_ = 0.74 *p* = 0.48
Diagnosis DLB/PD‐MCI/PDD/MCI‐LB	NA	9/10/3/26	20/3/13/5	FET *p* < 0.001
Male gender (%)	43/60 (71.67%)	41/48 (85.42%)	33/41 (80.49%)	FET *p* = 0.58
CAMCOG total	94.95 (7.11)	86.08 (11.88)	74.44 (14.83)	*t*_27.0_ = 2.35 *p* = 0.027
ACE‐R total	93.41 (4.06)	77.80 (14.39)	65.48 (17.70)	*t*_44.9_ = 2.87 *p* = 0.0063
UPDRS III total	2.16 (2.02) [0:9]	14.17 (8.24) [0:32]	25.07 (9.62) [2:42]	*t*_79.3_ = −5.69 *p* < 0.001
Taking levodopa	NA	18/48 (38%)	24/41 (59%)	FET *p* = 0.057
NPI hallucinations total score	NA	0.35 (0.79) [0.00:3.00]	3.00 (2.51) [0.00:8.00]	*t*_45.8_ = −6.41 *p* < 0.001
Angle task result	9.11 (2.98) [3.42:17.45]	25.14 (24.31) [5.21:89.49]	31.62 (27.33) [6.50:89.49]	*t*_65.1_ = −1.03 *p* = 0.31
Pareidolia: number of missed faces (max 8)	0.15 (0.37) [0:1]	0.39 (0.75) [0:3]	1.76 (2.14) [0:7]	*t*_22.8_ = −2.83 *p* = 0.01
Pareidolia: number of correct responses (max 40)	39.03 (1.29) [34:40]	36.95 (3.91) [27:40]	32.43 (5.96) [22:40]	*t*_29.7_ = 3.12 *p* = 0.004
Pareidolia: number of pareidolias	0.82 (1.21) [0:5]	2.66 (3.83) [0:13]	5.81 (5.03) [0:16]	*t*_33.1_ = −2.50 *p* = 0.018
ACE visuospatial (max 16)	15.61 (0.80) [13:16]	12.89 (2.69) [6:16]	9.96 (4.30) [1:16]	*t*_37.2_ = 3.01 *p* = 0.0047
CAMCOG praxis (max 12)	10.89 (1.56) [7:12]	9.31 (2.02) [5:12]	7.25 (1.73) [4:10]	*t*_23.8_ = 2.91 *p* = 0.007

*Note*: Values are mean (SD) [range] or *n*/*N* (%). For the pareidolia test, control *N* = 39, LB_noVH *N* = 38, LB_VH *N* = 21; ACE‐R control *N* = 41, LB_noVH *N* = 35, LB_VH *N* = 25; CAMCOG control *N* = 19, LB_noVH *N* = 13, LB_VH *N* = 16.

Abbreviations: ACE‐R, Addenbrooke's Cognitive Examination Revised; CAMCOG, Cambridge Cognitive Examination; DLB, dementia with Lewy bodies; FET, Fisher exact test; LB_noVH, Lewy body group without visual hallucinations; LB_VH, Lewy body group with visual hallucinations; MCI‐LB, mild cognitive impairment with Lewy bodies; PD‐MCI, Parkinson's disease with mild cognitive impairment; PDD, Parkinson's disease dementia; UPDRS, Unified Parkinson's Disease Rating Scale.

**FIGURE 1:**
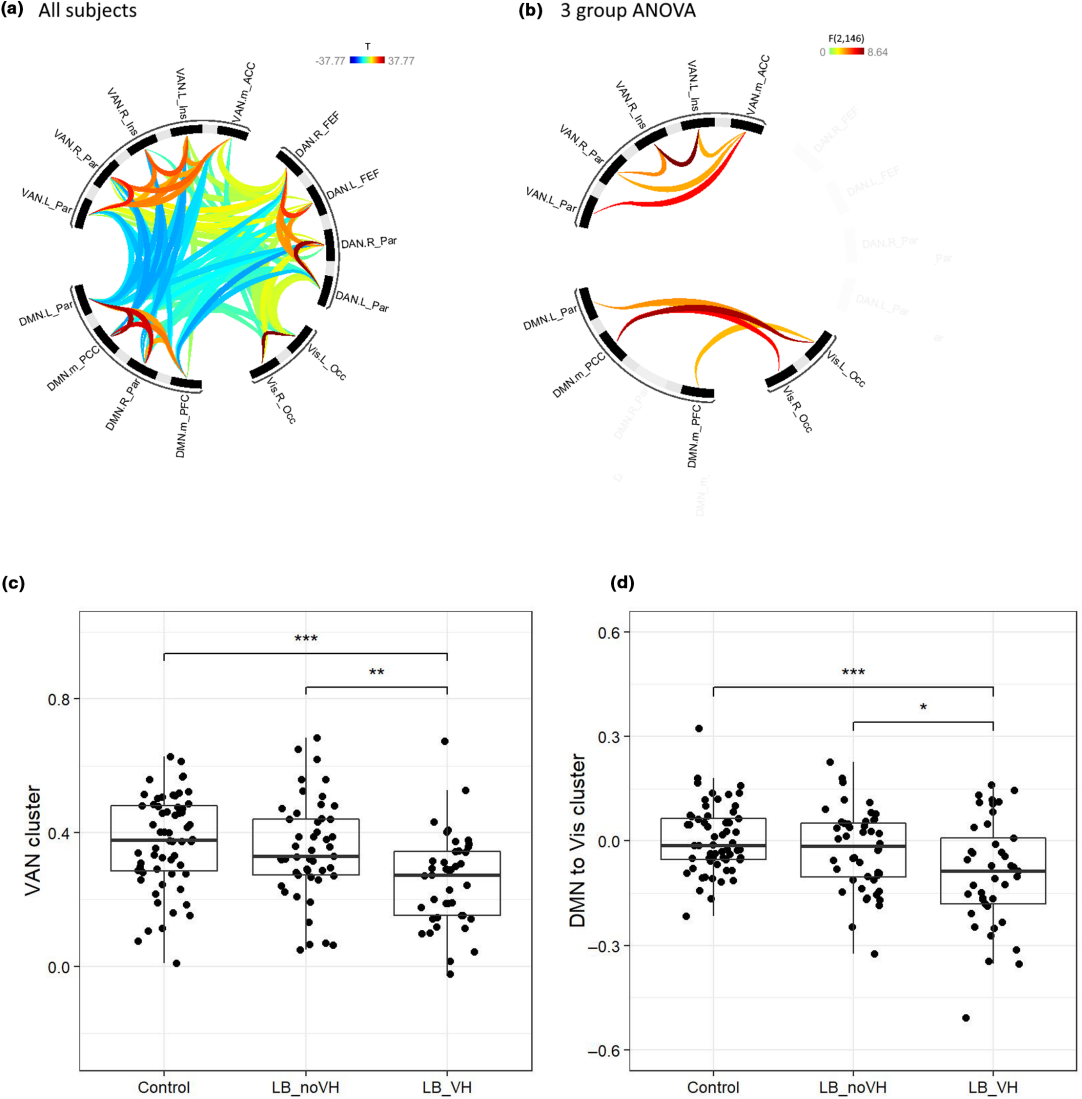
Functional connectivity from the Conn undirected fMRI analysis with hierarchical clustering: (a) connectivity strength across all participants; (b) group differences in connectivity; (c) connectivity in the VAN cluster identified on the group difference; (d) connectivity in the DMN to visual cluster identified on the group difference. ACC, anterior cingulate cortex; DAN, dorsal attention network; DMN, default mode network; FEF, frontal eye fields; Ins, insula; L, left; LB_noVH, Lewy body group without visual hallucinations; LB_VH, Lewy body group with visual hallucinations; m, medial; Occ, occipital; Par, parietal; PCC, posterior cingulate cortex; PFC, prefrontal cortex; R, right; VAN, ventral attention network; Vis, visual network.

With regard to functional connectivity, in keeping with previous reports on network connectivity, in all groups there was significant positive connectivity between regions in the same network, negative connectivity between DAN and DMN, and VAN and DMN, and positive connectivity between DAN and visual (Figures 1 and S3). In the DCM directed connectivity analysis there was negative effective connectivity from VAN to DMN and from DAN to DMN as well as (generally) positive connectivity between regions in the same network.

The functional connectivity analysis with hierarchical clustering found two sets of connections which were different in a three‐group ANOVA, between the DMN and visual, and also within the VAN network—left to right insula and the anterior to the more posterior VAN regions. In both cases, this was reduced in LB_VH compared to the LB_noVH and control groups (Figure 1). There were no changes in connectivity of the DAN. On DCM, the effective connectivity from the visual to the DMN regions in this cluster was significantly reduced in the LB_VH group compared to the control group (*t* = 2.08; *p* = 0.039) whilst the connectivity in the other direction from DMN to visual was not significantly different (*t* = −0.13, *p* = 0.9). There were no significant differences in effective connectivity between the more anterior and posterior regions in the VAN cluster.

After controlling in a linear regression for global cognitive *Z* score, the functional connectivity for the LB_VH was still significantly lower than for LB_noVH in both sets of connections (DMN:visual *t* = 2.27, *p* = 0.02; VAN *t* = 2.27, *p* = 0.025). For LB_VH versus control, the DMN:visual connectivity was still significantly lower (*t* = 2.67, *p* = 0.007) but the VAN connectivity was not significantly different controlling for cognition (*t* = 1.76, *p* = 0.08). The DCM effective connectivity was not significantly different between groups for any connections after controlling for cognitive score.

To investigate our specific hypotheses, connectivity between all of the regions within each network were averaged together and connectivity strengths were examined (1) within VAN and DMN, (2) between DMN and the VAN and visual networks and (3) of DAN to the other networks.

For the functional, undirected connectivity, in line with the hierarchical clustering analysis, decreased connectivity was found within the VAN in LB_VH versus control (*t* = 3.05, *p* = 0.003) and decreased connectivity within DMN:visual networks in LB_VH versus both the LB_noVH (*t* = 2.13, *p* = 0.035) and the control (*t* = 3.41, *p* = 0.0008) groups. There were no other significant differences, nor differences in the effective connectivity, although similar to the cluster analysis the visual to DMN connection was lower in the LB_VH versus control groups (*t* = 1.96, *p* = 0.057).

This analysis was repeated, controlling for UPDRS motor score and cognitive score separately. The DMN:visual connectivity was still significantly lower in the LB_VH versus control group after controlling for cognitive score (*p* = 0.0186) and with a trend for lower values in LB_VH versus LB_noVH (*p* = 0.064). The within VAN connectivity was not significantly different between groups after controlling for cognitive score, and neither the DMN:visual nor within VAN connectivity was significant after controlling for UPDRS.

**FIGURE 2:**
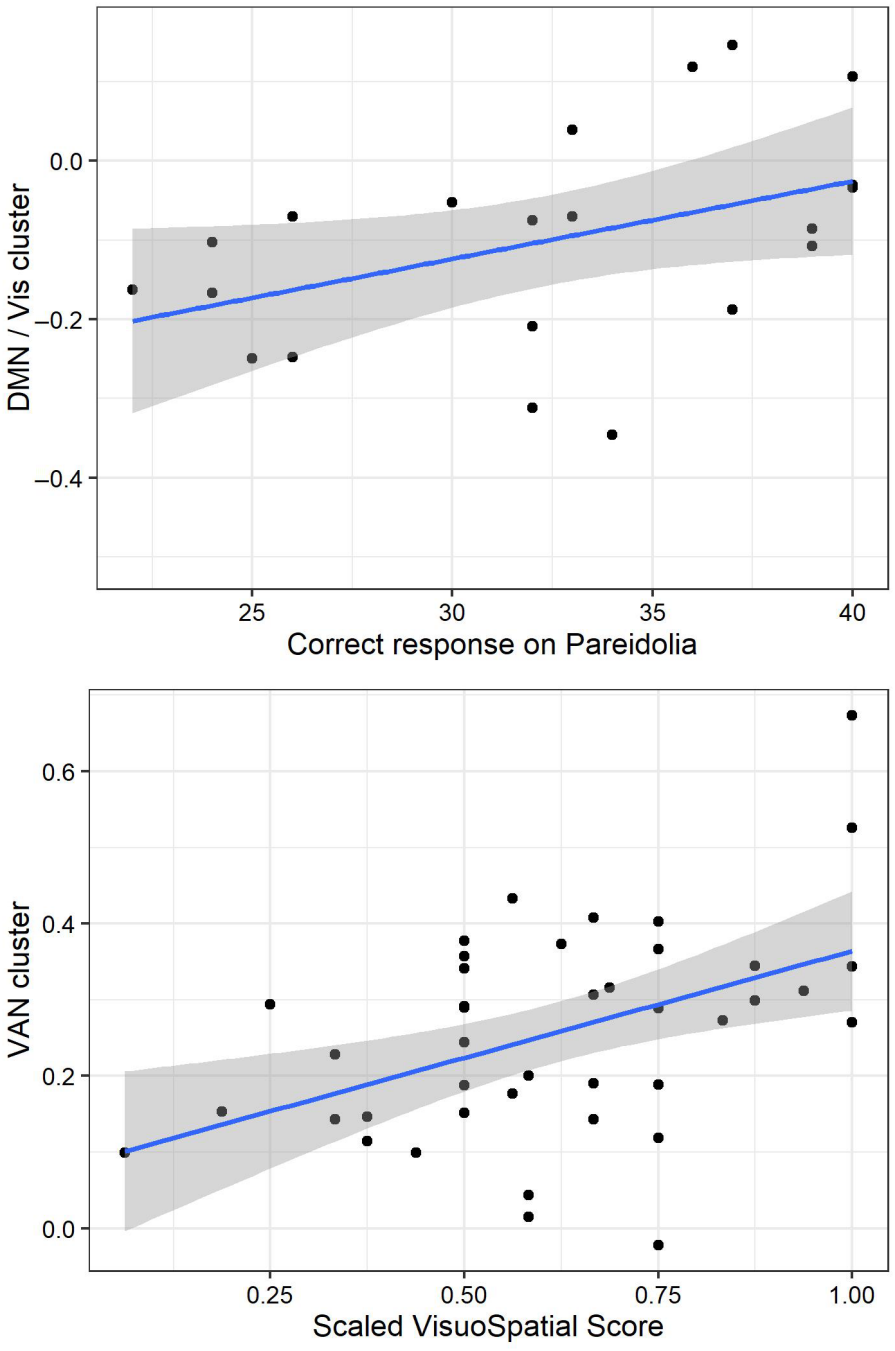
Top: Correlation of connectivity strength in the visual to DMN cluster with number of correct responses on the pareidolia noise task. Bottom: Correlation of connectivity strength in the VAN cluster with normalized visuospatial score. DMN, default mode network; VAN, ventral attention network; Vis, visual network.

For the two sets of significant connections in the hierarchical clustering analysis (VAN to VAN and visual to DMN), the correlation of functional connectivity strength with visual cognitive and neuropsychiatric scores within the LB_VH group was examined. There were no significant correlations for angle test (*N* = 34) or NPI hallucination subscore (*N* = 40), but there was a significant positive association of DMN:visual cluster functional connectivity with number of correct responses on the pareidolia task (*N* = 21, *r* = 0.435, *p* = 0.049). Additionally, the VAN to VAN cluster was correlated with the scaled visuospatial score from CAMCOG/ACE‐R (*r* = 0.459, *p* = 0.0026, Figure 2). The correlation with visuospatial score was still significant after controlling for total cognitive *Z* score (*p* = 0.017).

## DISCUSSION

Differences were found between LBD participants with versus without visual hallucinations within the VAN and between the DMN and the visual network, with the latter driven by reduced effective connectivity from the visual to the DMN network. These network alterations correlated with performance on visuospatial tasks. No differences in connectivity of the DAN were found.

The AN model [7] suggests that heightened arousal leads to upregulated VAN, along with increased activity in the DMN resulting in more self‐referential expectancies. During an hallucination, VAN and DMN integration with DAN, which would coordinate exogenous attention, is lost and increased coupling between the DMN and visual network allows false perceptions.

Our findings of decreased connectivity within the VAN is not in alignment with our hypotheses, or with the AN model. However, this model provides an explanatory framework for active hallucinations, whereas our participants were scanned resting with eyes closed (presumably not in a state of heightened arousal).

On the other hand, decreased connectivity within the VAN is consistent with the TDDMN model. Normally, the anterior insula and ACC (part of the VAN) are involved in switching brain activity between the resting DMN and the task positive exterior focused DAN [23, 24]. The TDDMN suggests that this relationship is altered in those with visual hallucinations, leading to decoupling of the DMN from attentional networks and increased influence of autobiographical priors. Our observation of decreased connectivity from the anterior insula and ACC within the VAN is supportive of this model. Decreased connectivity from the visual network to the DMN was also found, implying a reduced influence of visual stimuli modifying a priori expectations. The AN model predicts increased information flow from the DMN to the visual cortex leading to increased self‐referential imagery, but does not make any firm predictions about the influence of the visual cortex on the DMN. The TDDMN model suggests that due to reduced salience monitoring the DMN is more easily influenced by trivial visual stimuli, but again the model does not specifically make predictions regarding the connectivity of the visual cortex to the DMN.

After controlling for cognitive score, the connectivity in the VAN cluster of LB_VH group was still significantly different from that in the LB_noVH group, but not the controls. It is possible that there are changes in this network associated with cognitive decline, with perhaps compensatory increases in connectivity in early stage disease followed by decreases. It is also possible that altered connectivity in the LB_VH group might be due to poorer visual perception such that lack of sensory input leads to a decline in vison related functional activity.

Reports of visual cortex connectivity in visual hallucinations across the LBD spectrum have been somewhat conflicting. Yao et al. [25] found connectivity was higher in PD with visual hallucinations versus PD without visual hallucinations between the primary visual cortex and the insula and prefrontal cortex (but not posterior regions). The same group reported decreased connectivity between the hippocampus (part of the DMN) and several regions of the visual cortex in PD with visual hallucinations versus PD without visual hallucinations [26]. Diez‐Cirarda et al. [27] found decreased connectivity between visual and VAN in PD with versus without visual hallucinations, but do not report any alterations of the DMN connectivity. In DLB, decreased visual cortex connectivity [28] was reported with posterior cingulate (although the cluster is actually in the white matter) and increases with the post and precentral gyri. A study using DCM in PD [29] found reduced connectivity from the lateral geniculate nucleus to the visual cortex along with increased top‐down connectivity from prefrontal to visual cortex. The authors found increased connectivity from prefrontal cortex to visual, but did not examine the DMN. Finally, greater mind wandering was observed in PD with visual hallucinations versus PD without visual hallucinations [30], associated with increased occipital:DMN connectivity.

Metabolism in the occipital lobe has consistently been shown to be reduced in Lewy body disorders, particularly in those with cognitive impairment [31, 32, 33], and reduced metabolism is also present in parietal regions [34, 35]. However, the posterior cingulate cortex which is part of the DMN is relatively preserved in DLB [33, 35, 36] and thus our finding of reduced effective connectivity from the occipital lobe to the posterior DMN is in keeping with greater occipital versus posterior cingulate metabolic reductions.

An association between the number of correct responses on the pareidolia task and DMN:visual connectivity was found. The pareidolia task, which requires the presence or absence of faces in cloud‐like noise to be identified, is dependent on visual expectancies. It is therefore plausible that impaired connectivity of the primary visual cortex with the DMN results in less binding on the fundamental properties of a visual scene with autobiographical memories, leading to altered performance. In keeping with this, a previous study in PD with versus without visual hallucinations [37] found that participants with visual hallucinations were more dependent on a priori knowledge in a visual task.

**FIGURE 3:**
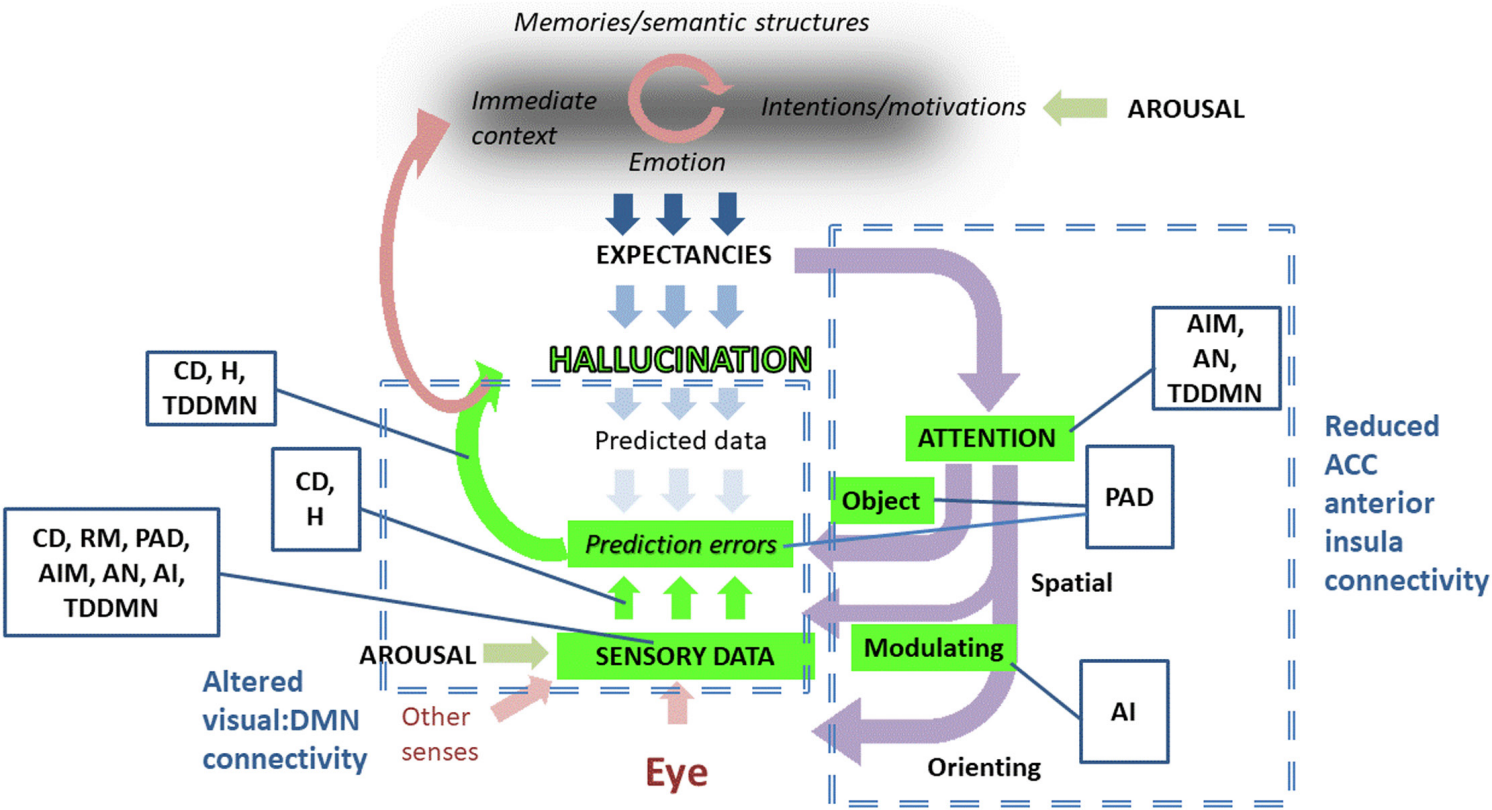
Suggested alterations to the hallucination framework evidenced by our findings shown in green. ACC, anterior cingulate cortex; AI, active inference; AIM, activation‐input‐modulation; AN, attentional networks; D, deafferentation; DMN, default mode network; H, hodological; PAD, perception and attention deficit; RM, reality monitoring; TDDMN, Thalamocortical Dysrhythmia Default Mode Network Decoupling. Adapted from Collerton et al. [5].

Given that our results are only partially consistent with network models of hallucinations, they have also been related to other approaches. A recently published consensus paper [5] proposes an integrated functional hallucination framework onto which the components of current hallucination models are mapped. As Figure 3 indicates, disruption between lower, purely visual areas and higher cognitive areas could potentially arise at a number of levels. Given the known occipital hypometabolism in DLB it is suggested that the altered visual to DMN connectivity that was identified primarily reflects a reduced influence of sensory data on higher visual function in people with LBD who hallucinate. The altered VAN connectivity may suggest additional impairments of processing within attentional shifting, such that salient visual features fail to reorient attention from hallucinatory perceptions.

Most of the eight current hallucination models synthesized in Collerton et al. [5] propose that hallucinations occur when poor vision or basic visual processing together with attentional impairments either lead to or occur in combination with biases towards expectation or mental imagery. Given that many of these models overlap, our data cannot unambiguously distinguish between them, particularly if considering individual cognitive functions. Thus, the pattern of disrupted connectivity across areas that has been identified here is consistent with active inference [38], activation‐input‐modulation [39], AN [7], deafferentation [40], hodological [41], perception and attention deficit [42] and TDDMN [8] models, at least in part. This inability to distinguish between models strongly suggests that these approaches need to be developed to have much greater precision before data can provide testable ways of distinguishing between them.

The strengths and weaknesses of our study are as follows. Our study incorporated a large number of patients with LBD with and without visual hallucinations, with a range of cognitive decline (from mild cognitive impairment to dementia) and phenotype (both PD and DLB). All participants were scanned with eyes closed; therefore connectivity patterns may not reflect those present during the eyes open condition, when visual processing of the external scene is occurring. In addition, no information is available on whether participants were experiencing visual hallucinations at the time of scanning, and instead the comparison is between individuals with versus without a tendency to hallucinations. Although combining studies allowed a larger group to be investigated, there were some differences in the cognitive tests used, which limited the analyses, and only a limited range of neuropsychological tests was available, particularly for visuoperceptual functions. It is difficult to disentangle the effects of cognition and disease severity from those of visual hallucinations. Frequency and severity of hallucinations tend to increase with worsening cognition and more severe PD and hence it is hard with a relatively small sample to distinguish the effect of disease severity from visual hallucinations on brain connectivity.

Overall, our findings suggest that disruption to the connectivity of the visual cortex plays a role in the aetiology of visual hallucinations, although further research utilizing image viewing or paradigms which attempt to recapitulate hallucinatory experience are needed to clarify the exact mechanism.

## AUTHOR CONTRIBUTIONS

**Michael J. Firbank:** Conceptualization; methodology; writing – original draft; formal analysis. **Daniel Collerton:** Writing – original draft; conceptualization. **Katrina daSilva Morgan:** Writing – review and editing. **Julia Schumacher:** Writing – review and editing. **Paul C. Donaghy:** Writing – review and editing. **John T. O'Brien:** Writing – review and editing; conceptualization. **Alan Thomas:** Writing – review and editing; conceptualization; funding acquisition.

## FUNDING INFORMATION

This work was supported by Alzheimer's Research UK, the NIHR Newcastle Biomedical Research Centre (BRC) based at Newcastle upon Tyne Hospitals NHS Foundation Trust and Newcastle University. J.T.O. is supported by the NIHR Cambridge Biomedical Research Centre. PCD is supported by the Medical Research Council (grant number MR/W000229/1).

## CONFLICT OF INTEREST STATEMENT

No authors have anything to declare relating to this manuscript.

## FINANCIAL DISCLOSURES

MJF: None. JS: None. DC: Publishing royalties from Wiley Blackwell. KdaSM: None. PCD: Grant funding from the MRC, Alzheimer's Society, Alzheimer's Research UK and the Lewy Body Society. He has received honoraria (paid to institution) for educational presentations for the Neurology Academy. JTO: Consultant for TauRx, Novo Nordisk, Biogen, Roche, Lilly and GE Healthcare and received grant or academic support from Avid/Lilly, Merck and Alliance Medical. AT: Grant funding from MRC, ESRC, NIHR, Alzheimer's Society, ARUK, PUK and ABBUK. JPT: Speaker fees for GE Healthcare, consultancy for EIP Pharma and royalties from Oxford University Press.

## Supporting information


Appendix S1

## Data Availability

The data supporting the findings of this study are available on the basis of a formal data sharing agreement and depending upon data usage, agreement for formal collaboration and co‐authorship, if appropriate. The AMPLE imaging data are available on request via the DPUK portal https://portal.dementiasplatform.uk/CohortDirectory/Item?fingerPrintID=AMPLE.
