# Supplementary material for: Functional connectivity in Lewy body disease with visual hallucinations
Source: Eur J Neurol. 2023 Nov 1;31(2):e16115. doi: 10.1111/ene.16115 (PMC11235993; doi:10.1111/ene.16115)
Supplement: Supplementary file 1 — Appendix S1 [file ENE-31-e16115-s001.docx]

# Functional connectivity in Lewy body disease with visual hallucinations

Michael J. Firbank, Daniel Collerton, Katrina daSilva Morgan, Julia Schumacher, Paul C. Donaghy, John T. O’Brien, Alan Thomas, John-Paul Taylor

Supplementary figure 1. Regions of interest in the analysis Ventral Attention Network (VAN) regions are in green colours, Dorsal Attention Network (DAN) in blue colours, Default Mode Network (DMN) regions in red colours, Visual network in yellow a) axial slice with parietal and posterior cingulate DMN, and (more anteriorly) VAN parietal region b) Sagittal slice with (anterior to posterior) DMN pre frontal cortex, VAN anterior cingulate cortex, DMN posterior cingulate cortex, visual occipital c) DAN frontal eye fields, and (posteriorly) parietal regions d) VAN anterior cingulate and insula regions.


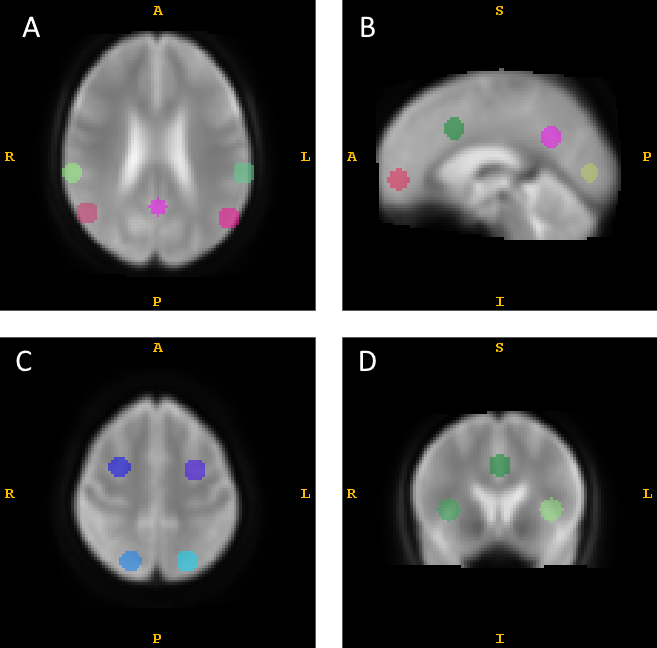


Supplementary figure 2. Quality scores from Conn preprocessing for the three groups.


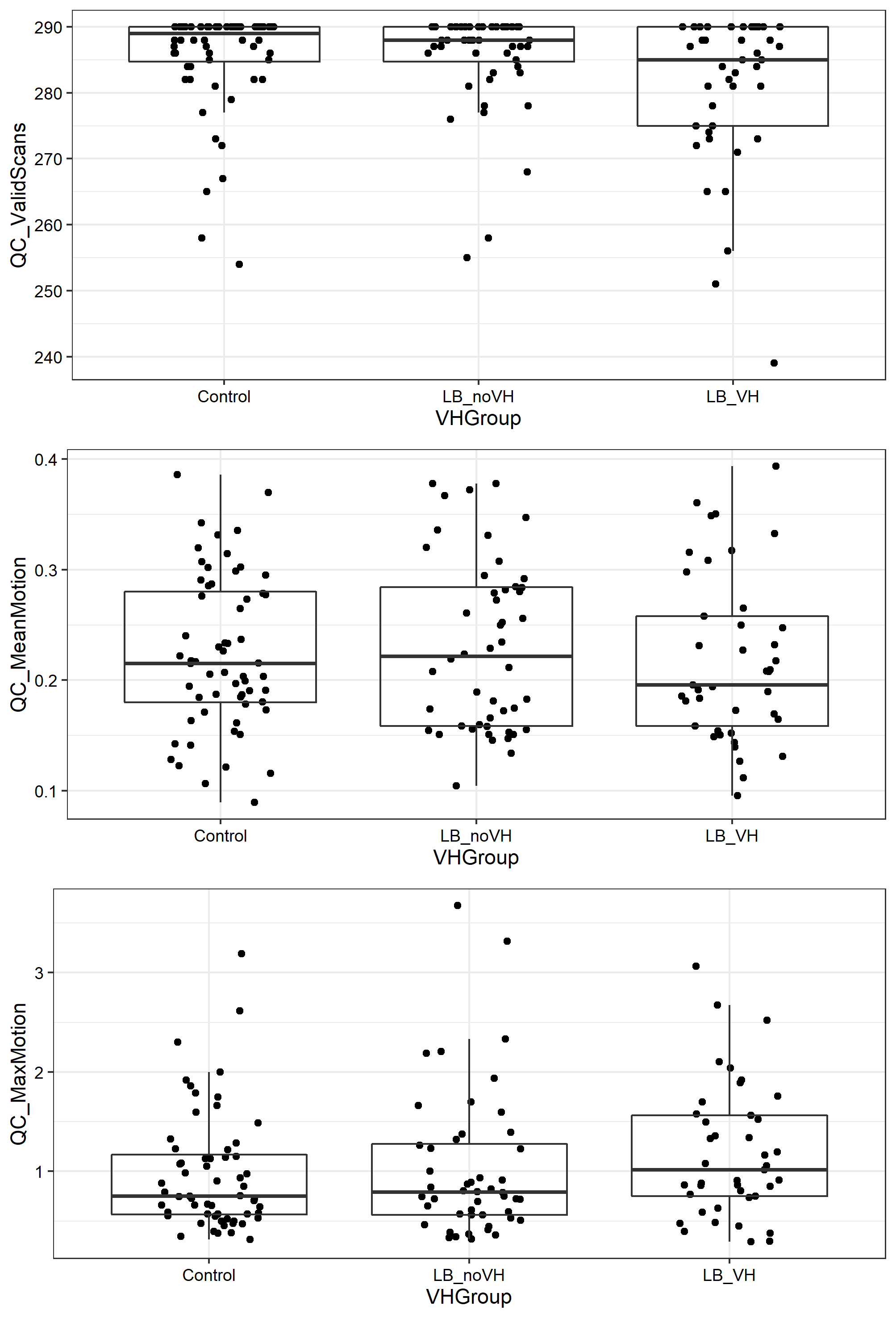


Supplementary Figure 3. Matrix shows Conn and DCM results thresholded at p < 0.05. Colour shows the connectivity strength. DAN, dorsal attention network; DMN, default mode network; VAN, ventral attention network; ACC, anterior cingulate cortex; FEF, frontal eye fields; Ins, insula; Occ, occipital; PCC, posterior cingulate cortex; PFC, pre-frontal cortex; Par, parietal; L, left; m, medial; R, right. LB_noVH, Lewy body group without visual hallucinations; LB_VH, Lewy body group with visual hallucinations.


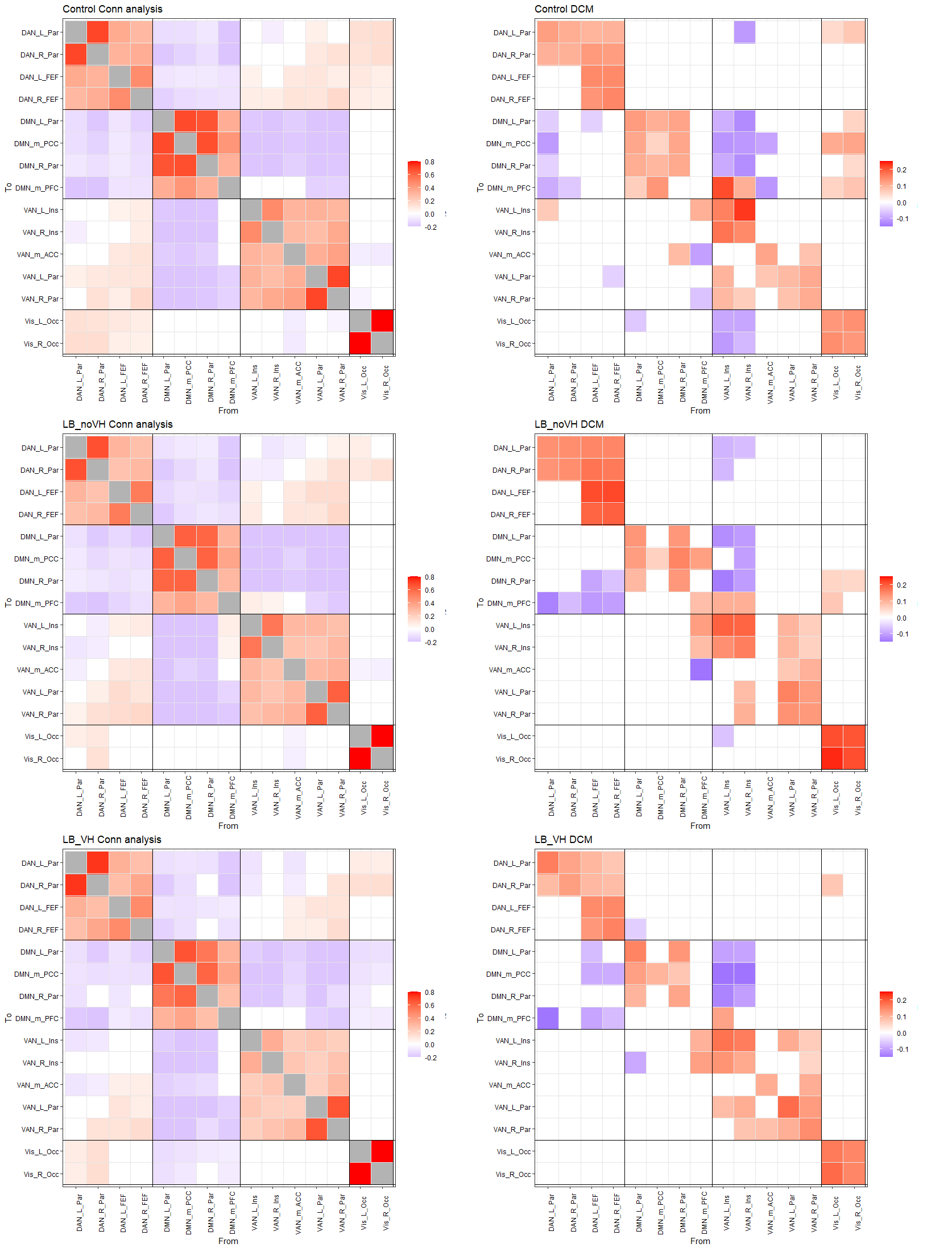


Supplementary table S1. Details of the Lewy body disease participants in the analysis, split according to diagnosis

|  | DLB [29] | Prob. MCI-LB [31] | PDD [16] | PD-MCI [13] | Stats |
| --- | --- | --- | --- | --- | --- |
| Age | 76.1 (7.1) | 74.5 (6.7) | 75.8 (3.2) | 72.3 (5.6) | F_3,85_=1.26 p=0.29 |
| Male | 23/29 (79.3%) | 28/31 (90.3%) | 13/16 (81.2%) | 10/13 (76.9%) | p=0.55 |
| Presence of visual hallucinations | 20/29 (69.0%) | 5/31 (16.1%) | 13/16 (81.2%) | 3/13  (23.1%) | p=1.6e-06 |
| Taking levodopa | 11/29 (37.9%) | 2/31 (6.5%) | 16/16 (100.0%) | 13/13 (100.0%) | p=2.3e-14 |
| Ldopa dose mg | 381.8 (205.6) | 450.0 (212.1) | 813.7  (435.2) | 590.4  (341.8) | F_3,38_=3.47 p=0.025 |
| Cholinesterase Inhibitor use | 27/29 (93.1%) | 15/30 (50.0%) | 9/16  (56.2%) | 0/13  (0.0%) | p=1.4e-08 |
| Antipsychotic  use | 2/29 (6.9%) | 0/30 (0.0%) | 3/16 (18.8%) | 0/13 (0.0%) | p=0.043 |
| StdGlobalCog | -7.9 (4.2) | -2.8 (2.5) | -4.5 (2.7) | -1.2 (1.2) | F_3,85_=19.75 p=8.4e-10 |
| UPDRS III Total | 22.6 (9.0) | 12.0 (8.0) | 27.8 (8.5) | 18.2 ( 9.6) | F_3,85_=14.08 p=1.6e-07 |
| CAMCOG Total | - | - | 71.8 (14.7) | 89.3 (6.7) | F_1,27_=15.73 p=0.00048 |
| ACE-R Total | 62.2 (16.6) | 82.5 ( 9.8) | - | - | F_1,58_=33.52 p=3e-07 |
| NPI Hallucinations Total Score | 2.5 (2.8) | 0.6 (1.1) | 2.6 (2.4) | 0.5 (0.9) | F_3,82_=6.92 p=0.00033 |
| NPI Hallucinations Severity Score | 1.0 (0.8) | 0.4 (0.7) | 1.0 (0.6) | 0.3 (0.5) | F_3,82_=5.49 p=0.0017 |
| Pareidolia: number of correct responses (max 40) | - | 36.5 (4.2) | 31.2 (5.2) | 37.8 (4.6) | F_2,56_=9.15 p=0.00037 |

Supplementary table S2. Medication use by diagnosis and VH group

|  | noVH | VH | Fisher’s exact test |
| --- | --- | --- | --- |
| Dementia |  |  |  |
|  |  |  |  |
| Cholinesterase inhibitors | 9/12 (75%) | 27/33 (82%) | P = 0.68 |
| Antipsychotic | 0/12 (0%) | 5/33 (15%) | P = 0.3 |
| Ldopa | 7/12 (58%) | 20/33 (61%) | P=1 |
|  |  |  |  |
| Mild Cognitive Impairment |  |  |  |
|  |  |  |  |
| Cholinesterase inhibitors | 10/35 (29%) | 5/8 (63%) | P = 0.10 |
| Antipsychotic | 0/35 (0%) | 0/8 (0%) | - |
| Ldopa | 11/36 (31%) | 4/8 (50%) | P=0.41 |

Supplementary table S3. Details of visual hallucinations for the LB_VH participants who completed the North East Visual Hallucinations Inventory (NEVHI).

Questions on the NEVHI are

1. Tricks “Do you feel like your eyes ever play tricks on you? Have you ever seen something that other people could not see?”
2. Object “Have you ever looked at an object or pattern and something else suddenly appeared or disappeared?”
3. Presence “Have you ever had the feeling of the presence of somebody, or something, in the corner of your eye?”
4. Shadow “Have you ever seen somebody or something, like a shadow, in the corner of your eye?”
5. Other “Have you ever had other visual experiences?”

| **Diagnosis** | **Age** | **Tricks** | **Object** | **Presence** | **Shadow** | **Other** | **NEVHI description** |
| --- | --- | --- | --- | --- | --- | --- | --- |
| MCI-LB | 79 | 1 | 1 | 1 | 1 | 0 | People on sofa; faces coming out of cushions; feels (sense of presence) may be due to seeing sides of glasses |
| MCI-LB | 71 | 1 | 0 | 0 | 0 | 1 | Not currently but was experiencing visual hallucinations at night |
| MCI-LB | 78 | 1 | 0 | 1 | 0 | 0 | People in the room - family members, speaks to them, no response; presence - something goes across vision |
| MCI-LB | 78 | 1 | 0 | 1 | 1 | 0 | Sense sombody hanging around on R shoulder; Non-distinct shadows on the L |
| MCI-LB | 73 | 1 | 0 | 0 | 1 | 1 | Seen men with white caps walking by the house; shadow - possibly tall lady in the room; black spots |
| PDD | 76 | 1 | 0 | 0 | 0 | 0 | People in his house having a party, a girl on the bed or standing next to him in the kitchen |
| PDD | 75 | 1 | 1 | 0 | 0 | 0 | sees people sitting in cars outside the house & a flower lady at the bottom of the stairs, lots of children playing in the garden |
| PDD | 71 | 1 | 1 | 1 | 0 | 1 | People in the house, at church, in garden, maybe complete or in shadow, hears low voices, pet types of animals, misperceptions especially when travelling by car, trees shaped into objects and moving, faces look different colours |
| PDD | 72 | 1 | 0 | 0 | 1 | 0 | Grey balls of fluff scuttling across the floor, a previously owned cat, single episode of a girl, shadowy blobs to L side, hears phone ringing when it is not. |
| PDD | 80 | 1 | 0 | 1 | 1 | 0 | dogs, people she knows, can happen anywhere in & out of the home, sometimes people talk to her & she answers, faces in the trees |
| PDD | 77 | 1 | 1 | 1 | 0 | 0 | Sees family members, former work colleagues, silent, occasional unpleasant sights, patterns in curtains like Tom & Jerry, perspective changes |
| PDD | 77 | 1 | 0 | 0 | 0 | 0 | Aware of vivid dreams which continue once he wakes up, often related to what ha sbeen on TV, has seen burglars trying to enter house |
| PD-MCI | 79 | 1 | 1 | 1 | 1 | 0 | Graves with people in long dresses rounde them, deceased husband, small children out of the window, a head coming through the curtailns, black things like mice running over the carpet, christmas tree turned into a little girl in a pink dress |
| PDD | 83 | 1 | 0 | 1 | 1 | 0 | presence and shadows occasionally, has seen deceased husband and shadowy figures |
| PDD | 72 | 1 | 1 | 1 | 1 | 0 | a man sitting in the chair dressed as a fisherman, she would not sit in that chair. Wallpaper moving around, figures in the lounge all silent, only occurs at her home |
| PDD | 73 | 1 | 1 | 0 | 0 | 0 | sees his deceased father, sees children top half only are in the bed, on the sofa, sitting on the wife's lap, sometimes recognises his son when he was a child, silent, not moving, distorted faces. Cushion has faces on it, patterns change into animals, leaves mistaken for birds |
| PD-MCI | 80 | 1 | 0 | 1 | 0 | 0 | Sees things out of the corner of his eyes in shadow, sees people standing by the door, strangers, formed but in shadow, hears his wife's voice |
| PDD | 77 | 1 | 0 | 1 | 0 | 0 | Beetles scuttling along, strangers in the bedroom, shadow like people, happens at night, presence feelings frequently |
| PD-MCI | 72 | 0 | 1 | 0 | 1 | 0 | Faces and things moving in wallpaper and carpets, mistakes trees for people when outside house, grey shadow in periphery on R side vanishes when he turns his head |
| PDD | 77 | 1 | 0 | 0 | 1 | 0 | People working in the house usually in the evening, people sitting on the sofa so he does not sit there, top half of bodies with distorted faces,black blobs which float past and disappear |
| PDD | 79 | 1 | 1 | 1 | 0 | 0 | Occasional outline of an adult or domestic animal, silent and walk away from him, people are in shadow, animals are more formed. Occurs inside the house, usually downstairs |
